# Extended Survival in a Dog with Primary Bone Hemangiosarcoma Following Treatment with Neoadjuvant Oncolytic Virotherapy and Standard of Care

**DOI:** 10.3390/vetsci12100921

**Published:** 2025-09-23

**Authors:** Courtney Labé, Andrea Chehadeh, Amber Winter, Sara Pracht, Kathy M. Stuebner, Mitzi Lewellen, Bishoy Eskander, M. Gerard O’Sullivan, Alexandru-Flaviu Tabaran, Christopher Ober, Michael S. Henson, Davis Seelig, Steve J. Russell, Jaime F. Modiano, Shruthi Naik, Kelly M. Makielski

**Affiliations:** 1 Department of Veterinary Clinical Sciences, College of Veterinary Medicine, University of Minnesota, St. Paul, MN 55108, USA; clabe@vet.upenn.edu (C.L.);; 2 Clinical Investigation Center, College of Veterinary Medicine, University of Minnesota, St. Paul, MN 55108, USA; 3 Animal Cancer Care and Research Program, University of Minnesota, St. Paul, MN 55108, USA; 4 Department of Veterinary Population Medicine, College of Veterinary Medicine, University of Minnesota, St. Paul, MN 55108, USA; 5 Masonic Cancer Center, University of Minnesota, Minneapolis, MN 55455, USA; 6 Department of Molecular Medicine, Mayo Clinic, 200 First Street SW, Rochester, MN 55905, USA; 7 Vyriad, Inc., 2900 37th St NW, Rochester, MN 55901, USA; 8 Department of Laboratory Medicine and Pathology, Medical School, University of Minnesota, Minneapolis, MN 55455, USA; 9 Center for Immunology, University of Minnesota, Minneapolis, MN 55455, USA; 10 Stem Cell Institute, University of Minnesota, Minneapolis, MN 55455, USA; 11 Institute for Engineering in Medicine, University of Minnesota, Minneapolis, MN 55455, USA; 12 Center for Genome Engineering, University of Minnesota, Minneapolis, MN 55455, USA

**Keywords:** cancer, dog, canine, hemangiosarcoma, virotherapy, doxorubicin, immunotherapy, osteosarcoma, comparative oncology

## Abstract

A three year old male neutered mixed breed dog presented with a mass on the right carpus and accompanying lameness. A Jamshidi bone biopsy was performed, and histopathology results were consistent with a sarcoma. The dog received oncolytic virotherapy (OV) with vesicular stomatitis virus (VSV) as part of a clinical trial in dogs with osteosarcoma (OSA). Ten days after VSV treatment, the affected limb was amputated, and histopathology was consistent with intramedullary HSA. Considering the new diagnosis, standard doxorubicin chemotherapy was prescribed. With this combination of therapies, the dog had an extended survival of more than seven years and remains alive at the time of writing. This is the first case report documenting OV given in conjunction with the standard of care for canine appendicular HSA.

## Simple Summary

Malignant bone cancer carries a grave prognosis in dogs despite treatment with surgery and chemotherapy. Neoadjuvant systemic oncolytic virus therapy has the potential to improve outcomes in some patients by stimulating an anti-tumor immune response. This case report presents extended survival in a five year old mixed-breed dog with primary bone hemangiosarcoma (HSA) treated with oncolytic virus therapy, amputation, and chemotherapy. According to the reviewed literature, this is the first reported case of a dog with HSA of the bone treated with oncolytic virus therapy.

## 1. Introduction

Hemangiosarcoma (HSA) is a highly aggressive vascular tumor arising from hematopoietic progenitor cells in the bone marrow [1] and accounts for approximately 2% of canine bone neoplasia [2,3]. Osteosarcoma (OSA) is the most common canine bone neoplasia, accounting for approximately 76 to 84% [2,4] of canine bone tumors. The telangiectatic osteosarcoma (tOSA) subtype is histologically similar to HSA, with factor VIII-related (FVIIIra) immunoreactivity in HSA most commonly used to distinguish between the two [5].

OSA and HSA of the bone both carry a guarded prognosis. A recent study comparing tOSA and appendicular HSA found a median overall survival (OS) of 299 days for dogs with appendicular HSA treated with chemotherapy in addition to amputation or radiation, and a median OS of 213 days for dogs with tOSA treated with chemotherapy in addition to surgery [6]. Neoadjuvant systemic oncolytic virotherapy (OV) has the potential to improve patient outcomes, and the VSV Immunotherapy and Genomics of Osteosarcoma Research (VIGOR) study was developed to test the safety, efficacy, and immunomodulatory effects of neoadjuvant intravenous vesicular stomatitis virus expressing interferon-beta and the sodium iodide symporter (VSV-IFNβ-NIS) therapy in dogs with localized appendicular OSA [7]. In the present report, we describe a dog that received VSV-IFNβ-NIS as part of the VIGOR clinical trial after an initial diagnosis of sarcoma of the bone, presumed to be OSA. Following amputation, histopathology confirmed HSA of the bone. After standard of care chemotherapy for HSA, the dog achieved a survival time of more than seven years and is currently alive at the time of writing. Currently, this is the only report of canine appendicular HSA treated with OV in addition to standard of care. The objective of this case report is to present the clinical, imaging, and histopathological findings, in addition to a discussion of treatment and the dog’s long-term survival.

## 2. Case Description

**Figure 1:**
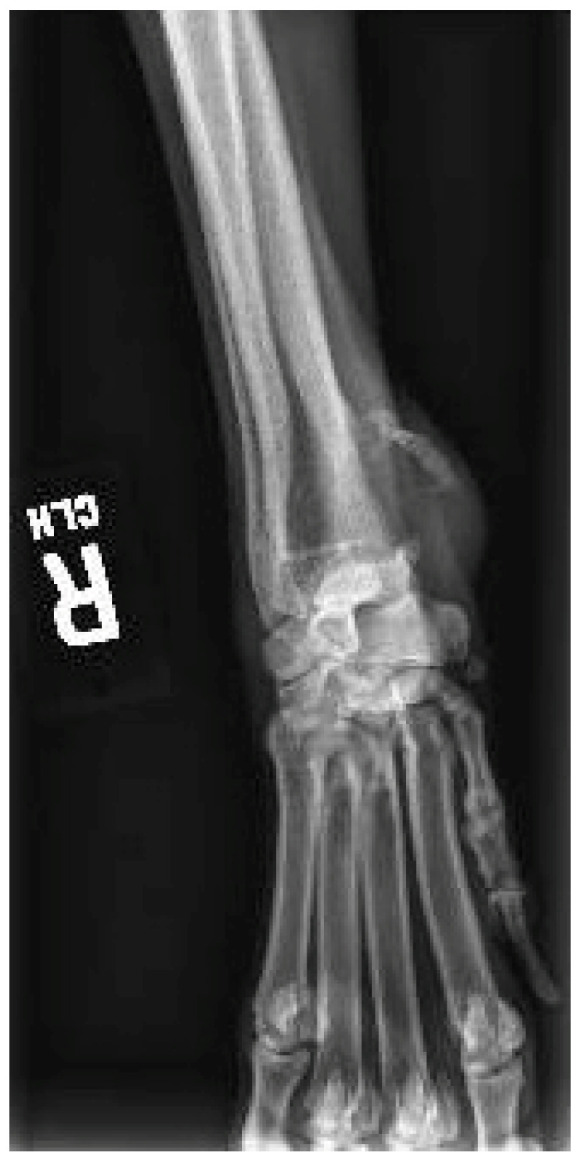
Right carpal radiograph of the mass and associated bony lesion.

An approximately three year old neutered male mixed-breed dog was presented to the Small Animal Surgery department at the University of Minnesota Veterinary Medical Center for evaluation of a right distal antebrachial mass. The mass had been present for one month with progressive growth. In the past, the dog had been hit by a car twice; however, it did not have a history of trauma to the affected limb. The dog also did not have a history of irradiation exposure. On presentation, an approximately 3 cm firm mass was present over the right distal radius and appeared to be fixed to the underlying tissue/bone. The dog had grade II/IV lameness in the right forelimb with moderate muscle atrophy, and a marked pain response was elicited upon flexion of the right carpus. Initial radiographs (Figure 1) of the right carpus showed focal cortical and peripheral medullary lysis of the craniomedial radius at the level of the distal metaphysis and epiphysis with severe focal extracapsular soft tissue swelling. Initial thoracic radiographs showed a mild bronchial pattern without evidence of intrathoracic neoplasia. Initial bloodwork was unremarkable. Fine needle aspirates of the mass were consistent with mesenchymal cell proliferation. A Jamshidi biopsy of the distal right radial mass was performed, and microscopic analysis showed the mass was composed of spindle to stellate shaped cells forming vague fascicles with round to oval nuclei with moderate anisokaryosis, coarsely clumped chromatin, and typically one to two moderately prominent nucleoli. Twenty-two mitotic figures were observed within 10 high powered fields. There were multiple areas of necrosis and hemorrhage. Definitive osteoid production was not identified, and no significant intratumoral inflammation was noted. The diagnosis was consistent with sarcoma, likely a primary bone sarcoma.

Based on the Jamshidi bone biopsy diagnosis of sarcoma and the likelihood of OSA, the owner elected to enroll the dog in the VIGOR study to receive VSV-IFNβ-NIS in addition to standard of care for OSA. The dog underwent a computed tomography (CT) scan of the thorax and abdomen as part of the study screening, and the CT showed no evidence of thoracic or abdominal primary or metastatic neoplasia. There were no lytic bone lesions of the axial skeleton noted on the CT scan. The dog received one dose of intravenous VSV-IFNβ-NIS (1 × 10^9^ TCID_50_/kg) without complications. Ten days following virotherapy, the dog’s right forelimb was amputated, and the dog was scheduled to begin six carboplatin treatments.

**Figure 2:**
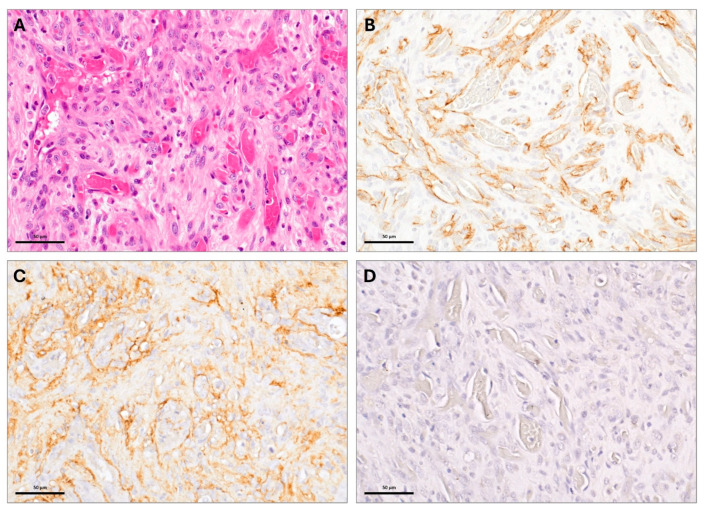
Histological staining of tumor sample following amputation. (**A**) H and E-stained section. Note the numerous, irregularly shaped and oriented vascular channels lined by slightly atypical endothelial cells. (**B**) Immunohistochemical staining for CD31. The previously noted vascular channels are lined by cells demonstrating moderate, brown anti-CD31 immunoreactivity indicative of an endothelial cell origin. (**C**) Immunohistochemical staining for FVIIIra. The previously noted vascular channels are lined by cells demonstrating mild to moderate, brown anti-FVIIIra immunoreactivity indicative of an endothelial cell origin. (**D**) Immunohistochemical staining for osteocalcin. Note the lack of anti-osteocalcin (brown) immunoreactivity, which supports a non-osteoblast origin to the neoplastic cells.

Following amputation, histopathologic analysis of an H and E-stained section of the tumor (Figure 2A) showed a partially necrotic neoplastic mass composed of a loose sheet-like arrangement of neoplastic spindle cells with oval to fusiform nuclei, with multiple small nucleoli and moderate amounts of eosinophilic cytoplasm. In many areas, the spindle cells lined irregular small spaces and clefts containing red blood cells and were occasionally separated by dense collagenous tissue. The neoplastic cells had invaded bone, with areas of bone tissue necrosis and reactive new bone tissue formation. Small fragments of necrotic bone tissue were sometimes bordered by macrophages and multinucleated giant cells. Mild periosteal and moderate intratumoral inflammation was noted. The tumor had a histologic appearance suggestive of an endothelial cell origin, due to the formation of clefts and irregular spaces containing red blood cells As definitive osteoid matrix production by the neoplastic cells was not observed, the initial diagnosis was sarcoma. Additional staining was performed to differentiate between HSA and OSA (Figure 2B–D). Uniformly across the neoplastic population, the cell surface was moderately to intensively positive for CD31 antigen (Figure 2B) and Factor VIII-related antigen (Factor VIIIra) (Figure 2C). The tumor cells were negative for osteocalcin (Figure 2D). Based on the histological findings, the positive immunoreactivity for CD31 and factor VIIIra, and the lack of osteocalcin, the initial histopathologic diagnosis of sarcoma was revised to intramedullary HSA.

**Figure 3:**
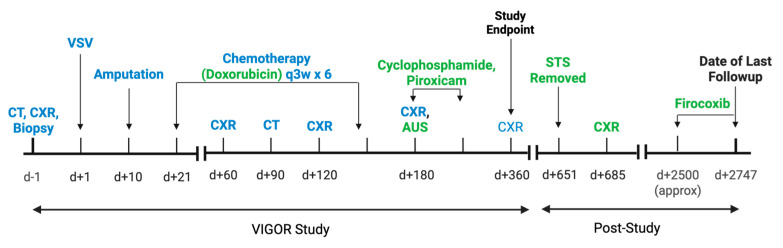
Timeline of key events in the medical history of a 3 year old neutered male mixed breed dog with intramedullary hemangiosarcoma during participation in the VIGOR Study and subsequent available follow-up period. The dog is reported to be clinically well dog at time of last follow-up, approximately 7 years after VSV treatment, and is now approximately 11 years old. Events included as part of the VIGOR Study are shown in blue, and events specific to only this case are shown in green. CT = computed tomography; CXR = chest x-rays; VSV = vesicular stomatitis virus; AUS = abdominal ultrasound; STS = soft tissue sarcoma.

Based on the diagnosis of bone HSA, the chemotherapy regimen was changed from carboplatin to doxorubicin. The dog received six doses of doxorubicin three weeks apart at the conventional dose of 30 mg/m^2^, with the exception of dose number four, which required a dose reduction to 27 mg/m^2^. Two months after the last doxorubicin dose, recheck thoracic radiographs and abdominal ultrasound were negative for metastasis. The dog began metronomic chemotherapy with 15 mg/m^2^ cyclophosphamide and 0.3 mg/kg piroxicam, with the cyclophosphamide eventually discontinued. The formal period of follow-up for the VIGOR Study was one year after treatment, after which continued diagnostic testing was at the discretion of the owner and the primary veterinarian. Approximately 17 months after the last dose of doxorubicin, the dog developed a grade I soft tissue sarcoma of the right eyelid that was incompletely excised. Approximately 18 months after the last dose of doxorubicin, which was almost two years after VSV treatment, the dog presented to the University of Minnesota Veterinary Medical Center emergency department for gastrointestinal signs and anorexia. Abdominal and thoracic radiographs taken at that time were negative for metastasis. At the time of writing, seven years after his initial treatment, the dog remains alive and is reported by the owner to be clinically performing well. The dog is reported to be receiving firocoxib (4 mg/kg once daily) for presumed osteoarthritis. Additional diagnostic testing has not been pursued. A timeline of key events in the VIGOR study and the patient’s available follow-up period is shown in Figure 3.

## 3. Discussion

Canine HSA is most commonly of visceral origin, and primary bone HSA is rare, with one retrospective study identifying 10 bone HSAs out of 1822 dogs diagnosed with HSA (0.5%) [8]. In this report, we describe a dog with primary bone HSA, initially presumed to be OSA based on a histopathologic diagnosis of sarcoma of the bone. The tOSA subtype is histologically and clinically similar to bone HSA, and this report highlights the utility of CD31, factor VIIIra, and osteocalcin immunostaining in the process of differentiating between intramedullary HSA and tOSA. Both HSA and OSA have cystic spaces histologically, with OSA characterized by the production of osteoid by malignant osteoblasts [9]. The tOSA subtype, however, has scant osteoid formation [10], impeding differentiation with the use of routine histopathology alone. In this case, ancillary osteocalcin, CD31, and FVIIIra immunostaining was performed. Osteocalcin protein is secreted by osteoblasts and expression can be utilized in the diagnosis of bone tumors, including tOSA [11,12]. In this case, osteocalcin staining was negative, consistent with a non-bone neoplasm. CD31, or platelet endothelial cell adhesion molecule-1 (PECAM-1) immunoreactivity has been utilized in the diagnosis of canine HSA [13]. Expression of Factor VIIIra has also been used to support the diagnosis of HSA. An analysis of 54 canine OSA tumors identified routine histopathology combined with FVIIIra to be reliable for the diagnosis of primary bone HSA [9]. One study of 122 canine splenic HSAs revealed variable CD31 and FVIIIra expression [14], highlighting the utility of multiple immunohistochemical stains to increase sensitivity when diagnosing primary bone HSA. In the present report, definitive osteoid production was not identified upon initial histopathology after a Jamshidi biopsy. Following amputation and histopathology, osteoid production once again was not observed and a histologic appearance suggestive of vascular endothelial cell origin was noted. The present report highlights the value of additional staining, namely CD31, Factor VIIIra, and osteocalcin to differentiate between intramedullary HSA and osteoid-devoid OSA.

A study comparing tOSA and appendicular HSA found dogs with bone HSA had a median progression-free survival of 101 days with local or local and systemic treatment [6], revealing the need for novel treatment options to improve outcomes. OV has been investigated in human angiosarcoma with variable success [15,16]. This dog experienced extended survival following treatment with standard-of-care therapy, including amputation of the affected limb followed by chemotherapy, in addition to treatment with OV, which might have initiated or amplified an anti-tumor immune response. The mild to moderate inflammation noted in the post-treatment amputation sample could potentially indicate an anti-tumor immune response; however, the pre-treatment tumor biopsy could have also contributed to the increased inflammation. As this dog was the sole OV recipient within the study that was diagnosed with intramedullary HSA, and continued monitoring with advanced imaging was not routinely performed beyond the timeframe of the clinical trial, we must be cautious interpreting the role of OV in the dog’s response. The relative contribution of each component of treatment (OV, amputation, chemotherapy) to this dog’s extended survival cannot be determined. Additionally, the NSAID (firocoxib) that the dog has been receiving for presumed osteoarthritis may have some anti-cancer effects that could be contributing to the dog’s extended survival. Nonetheless, this report highlights the success of an index dog receiving neoadjuvant OV followed by amputation and chemotherapy to treat intramedullary HSA, which may provide a foundation for additional studies to ascertain the safety and efficacy of this approach to treat an otherwise incurable cancer.

## Data Availability

The original contributions presented in this study are included in the article. Further inquiries can be directed to the corresponding author.
